# Incremental Net Benefit and Incremental Cost-Effectiveness Ratio of COVID-19 Vaccination Campaigns: Systematic Review of Cost-Effectiveness Evidence

**DOI:** 10.3390/vaccines11020347

**Published:** 2023-02-03

**Authors:** Giuseppe Santoli, Mario Cesare Nurchis, Giovanna Elisa Calabrò, Gianfranco Damiani

**Affiliations:** 1 Section of Hygiene, Department of Life Sciences and Public Health, Università Cattolica del Sacro Cuore, 00168 Rome, Italy; 2 Department of Woman and Child Health and Public Health, Fondazione Policlinico Universitario A. Gemelli IRCCS, 00168 Rome, Italy

**Keywords:** COVID-19, SARS-CoV-2, vaccination, cost-effectiveness, health policy, value, vaccination campaigns, vaccines

## Abstract

SARS-CoV-2 vaccination has been the most effective tool to prevent COVID-19, significantly reducing deaths and hospitalizations worldwide. Vaccination has played a huge role in bringing the COVID-19 pandemic under control, even as the inequitable distribution of vaccines still leaves several countries vulnerable. Therefore, organizing a mass vaccination campaign on a global scale is a priority to contain the virus spread. The aim of this systematic review was to assess whether COVID-19 vaccination campaigns are cost-effective with respect to no vaccination. A systematic literature search was conducted in the WHO COVID-19 Global literature database, PubMed, Web of Science, Embase, and Scopus from 2020 to 2022. Studies assessing the COVID-19 vaccination campaign cost-effectiveness over no vaccination were deemed eligible. The “Drummond’s checklist” was adopted for quality assessment. A synthesis of the studies was performed through the “dominance ranking matrix tool”. Overall, 10 studies were considered. COVID-19 vaccination was deemed cost-effective in each of them, and vaccination campaigns were found to be sustainable public health approaches to fight the health emergency. Providing economic evaluation data for mass vaccination is needed to support decision makers to make value-based and evidence-based decisions to ensure equitable access to vaccination and reduce the COVID-19 burden worldwide.

## 1. Introduction

The novel coronavirus disease 2019 (COVID-19) since December 2019 has claimed more than 6.66 million deaths [1], overwhelming health care systems around the world and leading to a global economic recession [2]. 

To respond to this health emergency, the scientific community made a strenuous endeavor to promptly develop a safe and effective vaccine, which drove the application of unexplored or nearly unexplored technologies [1]. The World Health Organization (WHO) has currently listed nine vaccines in the Emergency Use Listing (EUL) according to the latest evidence on safety, effectiveness, and sustainability [3]. 

The aforementioned vaccines can be divided into three categories based on the way they are designed [4,5]: (1) whole-microbe vaccines, incorporating the entire virus, which has been attenuated or inactivated by chemical or physical means including viral vector vaccines [6]; (2) recombinant vaccines, which lack the genetic material of the pathogenic microorganism, hence they incorporate only the viral proteins, protein segments, or subunits [7]; and (3) nucleic acid vaccines, based on the genetic material of the virus (RNA or DNA), encapsulated in lipid nanoparticles (LNPs), which can induce the receiver’s cells to produce the viral antigen [8]. 

To date, over 13 billion vaccine doses have been administered worldwide [1] and 69.1% of the world population has received at least one dose of a COVID-19 vaccine [9]. Even though these data seem encouraging, they fail to show the profound disparities between the developed and developing world. While high-income countries (HIC) have achieved an average vaccine coverage of 67%, only 25.9% of people in low-income countries (LIC) have received at least one dose [9]. Furthermore, some low-income countries, especially in Africa, have been predicted to not achieve 70% coverage until the end of 2024 [10].

A comprehensive and evidence-based insight of the most cost-effective immunization strategies and resource allocation is required, especially for resource-limited countries, to tackle the ongoing pandemic and to promote the preparedness and resilience of the health care systems to the epidemiological threats that the future may reserve. Economic evaluations are commonly used to evaluate both the economic and clinical value of medical technologies [11]. Most economic evaluations report their findings by adopting the incremental cost-effectiveness ratio (ICER), which is the ratio of the incremental costs (ΔC) and the incremental effect (ΔE) between the intervention and its alternative. According to the WHO guide for the standardization of economic evaluations of immunization programs [12], the incremental effect should be measured in natural units such as deaths averted, years-of-life saved (YLS), disability-adjusted life years (DALYs), quality-adjusted life years (QALYs), or quality-adjusted life days (QALD) [13,14]. By comparing the ICER of the intervention to a pre-established threshold, decision makers can determine whether it is opportune to pursue the implementation of the intervention, which is deemed cost-effective if the value of its ICER falls below the aforesaid threshold. However, the use of ICER can lead to statistical and interpretability problems given its nature of ratio measure [14]. The incremental net monetary benefit (INB) can be a viable option to elude the ordering problems of ICER. INB is expressed as the value of the incremental effect multiplied by a predetermined threshold less the incremental costs. In terms of the cost-effectiveness decision rule, the intervention is considered cost-effective when its INB is greater than 0 and not cost effective when it is not [15,16]. No secondary studies are currently available in the most searched scientific databases comparing vaccination to standard care, reporting results expressed as ICERs and/or INBs. 

Within the above context, the aim of this systematic review was to assess whether COVID-19 vaccination campaigns are cost-effective with respect to no vaccination.

## 2. Materials and Methods

### 2.1. Study Design and Search Strategy 

The research question was framed using the Population, Intervention, Comparison, Outcome (PICO) model, inquiring whether vaccinating the entire population against COVID-19 is more cost-effective than no vaccination, by using ICERs or INBs as composite measures. Articles were retrieved from the WHO COVID-19 Global literature database, PubMed, Web of Science, Embase, and Scopus from 2020 to May 2022. 

The search strategy followed the Preferred Reporting Items for Systematic Reviews and Meta-Analyses (PRISMA) checklist [17]. The Boolean search query, employed for the database search, was formulated by adopting medical subject headings (MeSH) and free-text words such as “vaccination”, “quality adjusted life years”, “QALY”, “cost-utility analysis”, “cost analysis”, “COVID-19”, and “SARS-CoV-2”. The Snowball method was used as a complementary tool to detect the articles that were not identified through a database search. 

### 2.2. Study Selection 

Eligibility criteria for the present systematic review were determined as follows: cost-effectiveness analyses, focused on the general population, comparing vaccination against COVID-19 to no vaccination, reporting QALYs as a measure of outcome, cost estimates for each alternative, and ICERs and/or INBs. Only articles written in English and available in full text were included. The ICER and/or INB, when available, were the outcomes of interest. 

The assessment for the inclusion criteria was conducted by two authors independently. It comprised two rounds: an initial screening, which consisted in the evaluation of the titles and abstracts of all the articles retrieved and a second screening, focused on the examination of the full text of each study selected in the first screening round, in order to determine the final eligibility. In the event of a disagreement, a third author was in charge of solving the disagreement by discussing the characteristics of the study based on the inclusion and exclusion criteria. 

### 2.3. Quality Assessment 

Quality assessment was conducted by two independent researchers, relying on Drummond’s checklist for assessing economic evaluations [18], to determine to what extent the included studies met the criteria for a reliable economic evaluation. Disagreements were solved by a third researcher. 

The aforesaid checklist consists of 10 items and evaluates the following elements: the research question; the description of the competing alternatives; the comprehensiveness of the identification of costs and consequences for each alternative; the study design; the appropriateness of the physical units chosen to measure the consequences; the credibility of the evaluation of costs and consequences; the presence of adjustment for differential timing of costs and consequences; the incremental analysis of costs and consequences; the presentation of results with uncertainty and sensitivity analyses; and the presentation and discussion of the study results. Researchers assigned “yes”, “no”, or “unclear” to each item. Then, according to the rating scale developed and proposed by Doran et al., a score was assigned to each assessed study. Based on the final score, the quality can be poor (i.e., 1–3 points), average (i.e., 4–7 points), or good (i.e., 8–10 points). 

### 2.4. Data Extraction 

Three independent authors performed the data extraction. An electronic data extraction form was designed including the study characteristics (i.e., journal, publication year, and country), type of economic evaluation, vaccine platform, type of simulation model, perspective, sample size, type of sensitivity analysis, total costs, total QALYs, and, if available, ICER, in the event of studies presenting different scenarios of the least cost-effective one (i.e., reporting the highest ICER), was considered for the narrative synthesis. 

### 2.5. Data Synthesis 

All costing components retrieved from the selected articles were adjusted to 2021 values through the consumer price index (CPI) and converted to 2021 international dollars (I$). CPIs and PPP values were collected from the International Monetary Fund website and from the OECD database, respectively [19,20]. 

A narrative synthesis of the included studies was conducted through the Joanna Briggs Institute (JBI) three by three “dominance ranking matrix tool” (DRM) [21]. The DRM allows decision makers to draw conclusions from the study results by grouping them into three categories: (1) favored, when the intervention is less costly and equally effective, less costly and more effective, or equally costly but more effective than its alternative; (2) rejected, when it is more costly and less effective, equally costly and less effective, or more costly and equally effective with respect of its comparator; and (3) unclear in any other scenario, in this case, decision makers ought to assess the advantages and disadvantages of the intervention and its alternatives and compare the cost per QALY to a predetermined threshold to determine whether to favor or reject the intervention. The WHO–CHOICE 3× gross domestic product (GPD) per capita threshold was adopted to assess the cost-effectiveness of unclear results from DRM [22].

## 3. Results

### 3.1. Study Selection 

**Figure 1:**
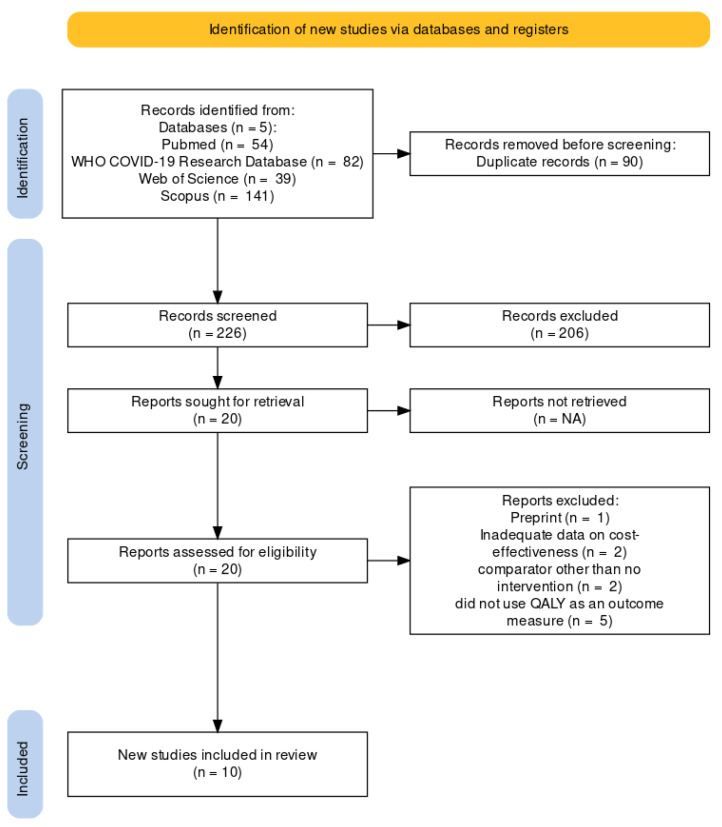
Study selection flow diagram.

The database search yielded a total of 316 publications, which were reduced to 226 after duplicate removal. Through title and abstract screening, twenty articles were selected for full-text examination. Full-text examination led to the exclusion of ten papers given the eligibility criteria. Therefore, ten articles were included in the systematic review [23,24,25,26,27,28,29,30,31,32]. Figure 1 depicts the study selection diagram.

### 3.2. Study Characteristics 

All selected articles were full economic evaluations, reporting the costs and effectiveness of vaccination campaigns compared with no vaccination. Nonetheless, different vaccine platforms were taken into account: one study considered two nucleic acid vaccines (i.e., Pfizer-Biotech/Comirnaty and Moderna) and an adenoviral vector vaccine (i.e., Vaxzevria) [23]; one study confronted an inactivated vaccine (i.e., CoronaVac) and two viral vector vaccines (i.e., Vaxzevria and Janssen) [30]; finally, one study focused on two inactivated vaccines (i.e., CoronaVac and BBIBP-CorV) [25], and the remaining articles made hypothetical assumptions on the effectiveness and cost components related to vaccination [24,26,27,28,29,31,32]. Seven papers assessed the cost-effectiveness of the intervention only using the ICER as an outcome measure [23,24,27,31], two reported the INB [26,32], and one study reported both of them [25]. The settings of the economic evaluations comprised 12 countries, which belonged to four of the six WHO regions [33]: the Region of the Americas (USA, Canada, and Brazil), the European Region (Spain, UK, Israel, and Turkey), the Southeast Asian Region (Thailand and Indonesia), and the West Pacific Region (China, Philippines, and Singapore). All of the included studies investigated the uncertainty associated with the input parameters through ad-hoc sensitivity analyses. Table 1 reports the additional characteristics for each of the included studies. 

### 3.3. Quality Assessment

Table 2 shows the quality of the studies according to Drummond’s 10-item checklist. Two studies totaled 6 points [27,28], whereas four papers scored 7 points [23,24,25,26,29], suggesting an average rating. Amongst the remaining studies, two totaled 8 points [31,32], while the other two achieved 9 points [26,30], implying a good overall quality.

**Table 1:** Additional study characteristics. Abbreviations: USD (United States dollar), DKK (Danish Krone), EUR (euro), CAD (Canadian dollar), ICER (incremental cost-effectiveness ratio), NMB (net monetary benefit).

Author, Year	Country	WHO Region	GDP Per Capita	Type of Vaccine	Perspective	Outcome Measure	Currency	Intervention Costs (I$)	Comparator Costs (I$)	Intervention Effects (QALYs)	Comparator Effects (QALYs)	ICER (I$/QALY)	NMB (I$)
Wang et al., 2021 [23]	Israel	EUR	39,481	mRNA (Comirnaty)	Health system Societal	ICER	USD	1,577,960	4,278,161	1,798,286	1,790,002	−326	Not reported
ibidem				mRNA (Moderna)				1,338,638	4,278,161	1,798,120	1,790,002	−362	
ibidem				Viral vector (Vaxzevria)				1,694,309	4,278,161	1,797,458	1,790,002	−347	
Debrabant et al., 2021 [24]	Denmark	EUR	61,063	mRNA	Health system	ICER	DKK	45,281,649.8	88,429,852.1	−714	−5410	8193.7–18,242.5	Not reported
Jiang et al., 2022 [25]	Hong Kong	WPR	46,324	inactivated	Societal	ICER/INB	USD	76,643,624	96,007,188	−181	−218	Cost-saving	45,379,143
ibidem	Indonesia	SEAR	3870					44,388,774	48,144,281	−544	−570		5,662,212
ibidem	PRC	WPR	10,435					20,625,625	23,056,317	−189	−205		8,645,824
ibidem	Philippines	WPR	3299					17,608,767	17,850,779	−925	−972		1,019,699
ibidem	Singapore	WPR	59,798					52,772,707	101,156,430	−202	−231		28,632,981
ibidem	Thailand	SEAR	7187					79,995,715	94,299,089	−3391	−3989		7,984,741
Sandmann et al., 2021 [26]	United Kingdom	EUR	41,059	Not stated	Health system	INB	GBP	215 × 10^9^	130 × 10^9^	−78,900,000	−93,100,000	Not reported	737.1 × 10^9^
Marco-Franco et al., 2021 [27]	Spain	EUR	30,116	Not stated	Health system	ICER	EUR	Not stated	Not stated	Not stated	Not stated	8565	Not reported
Hagens et al., 2021 [31]	Turkey	EUR	8536	Not stated	Health system Societal	ICER	USD	1,339,290,179	407,011,036	Not stated	Not stated	1250	Not reported
Kohli et al., 2021 [28]	United States	AMR	63,593	Not stated	Health system	ICER	USD	29.4 × 10^6^	21.3 × 10^6^	not stated	not stated	8476	Not reported
Kirwin et al., 2021 [32]	Canada	AMR	67,656	mRNA	Health system	NMB	CAD	Not stated	Not stated	not stated	not stated	Not reported	240.9 × 10^6^
Padula et al., 2021 [29]	United States	AMR	63,593	Not stated	Health system	ICER	USD	13.5 × 10^9^	34.9 × 10^9^	−0.879	−0.899	Cost-saving	Not reported
Fernandes et al., 2022 [30]	Brazil	AMR	7519	Inactivated (CoronaVac)	Health system	ICER	USD	121 per patient	88.55 per patient	0.87	0.869	17,758	Not reported
ibidem				Viral vector (Vaxzevria)				41.1 per patient	88.55 per patient	0.871	0.869	−23,161	
ibidem				Viral vector (Janssen)				77.8 per patient	88.55 per patient	0.87	0.869	−1690.8	

### 3.4. Vaccination Cost-Effectiveness 

Table 3 provides the results of the three-by-three DRM adopted to interpret the findings of the included studies. 

As shown by the DRM, vaccination is constantly cost-effective if compared with no vaccination. A total of six articles favored the vaccination campaign, as it was both less costly and more effective than no intervention. The cost-effectiveness of vaccination was deemed unclear by four studies, as it was more costly but also more effective than not vaccinating. 

Sandmann et al. [26] investigated the cost-effectiveness of vaccines against COVID-19 over no intervention at all as well as with respect to different mitigation scenarios by assuming the implementation of an initial lockdown, followed either by voluntary physical distancing or by mandatory physical distancing when the COVID-19 daily incidence reached predetermined thresholds. It also investigated the COVID-19 vaccination cost-effectiveness by varying the efficacy of the vaccine from 50% to 75%. The cost of COVID-19 in an unmitigated scenario and without vaccination was estimated at I$737.1 billion (95% UI 558.5–956.6) over a 10-year time horizon. INBs for a 75%-effective vaccine ranged from I$18.2 billion to I$506.5 billion across different physical distancing scenarios. The INB value varied significantly depending on the time of the introduction of the vaccines, decreasing as the beginning of the vaccination campaign was delayed. 

Kohli et al. [28] estimated an ICER at I$8476 to vaccinate the whole adult population if compared to no vaccination, and the cost per dose (which was assumed to be I$36.9, i.e., I$73.8 per course in the base case scenario), the vaccine efficacy against COVID-19 (60% in the base case scenario), and the prioritization scheme greatly influenced the cost per QALY of the vaccination campaign. It is worth noticing that the initial vaccine supply also had a significant impact on the outcome of the immunization campaign, which could prevent from 31% to 23% of expected deaths as the vaccine supply decreased. 

Both the above-mentioned studies highlighted the importance of a swift distribution of the vaccines 

Wang et al. [23] found an ICER of mass vaccination in Israel with Moderna, Pfizer, and AstraZeneca, ranging between −I$357 and −I$321 per QALD from a health care system perspective. The negative values of the ICER indicate that vaccination was cost saving with respect to no vaccination. The price per administration was estimated as $82 for Moderna, $48 for Pfizer, and $30 for AstraZeneca, for two doses. According to the authors’ findings, a mass vaccination campaign would reduce days of hospitalization up to 85% if mRNA vaccines (i.e., Moderna and Pfizer) are deployed, while 78% of days of hospitalization would be prevented by deploying AstraZeneca vaccines.

Debrabant et al. [24] calculated, through a differential equation model, an ICER between I$6647.7 and I$19,788.5 per QALY. Comparing COVID-19 vaccination of only the population aged ≥60 years to no vaccination, the cost for each vaccination course would range from I$46.4 to I$77.3. Vaccinating 70% of Danish population would lead to an ICER between I$21,798.3 and I$46,070.1 per QALY. Although mass vaccination would have a higher cost per QALY than a targeted vaccination for individuals aged ≥60 years, its ICER would decrease up until I$14,532.2 per QALY, if the productivity losses are included. The vaccine was assumed to have 100% effectiveness against COVID-19. Decreasing the vaccine’s effectiveness from 100 to 80% would lead to a decrement in the gained QALYs between 13 and 16%, an increase in total health care costs between 7 and 18%, and an increased productivity loss around 4–14%. Kirwin et al. [32] estimated an INB for mRNA vaccines at an I$25,000 cost-effectiveness threshold in the Alberta Province, Canada. Costs and gained QALYs varied significantly, depending on the prioritization scenarios, as the no prioritization scenario showed the highest INB, I$240.9 million versus I$205.7 million for the age and risk-based prioritization scenario. However, the former prevented fewer hospitalizations when compared with the latter of 3419 and 3432 respectively. 

Marco-Franco et al. [27] developed an ad hoc model, named the best adjustment of related values (BARV) method, able to minimize the possible errors of all the variables, by adding weighting to more reliable data and by an iteration process for the uncertain variables. Through the above method, the authors demonstrated that the immunization against COVID-19 was highly cost effective in Spain, estimating an ICER of I$8565.3 (8221.5–8805.6) for a 70% effective two-dose vaccine at the price of I$50 per dose.

Jiang et al. [25] examined the cost-effectiveness of vaccinating 50% of the population with inactivated vaccines in six different jurisdictions, namely, Hong Kong, Indonesia, Mainland China, Philippines, Singapore, and Thailand per every 100,000 vaccinated individuals, the ICER would be I$−382,729; −53,553.6; −7608.7; −97,706.9; −190,420; −69,363.2, respectively. Vaccine efficacy against COVID-19 transmission and severe disease were estimated using meta-analyses of phase 3 trials of inactivated vaccines. In the simulations, the vaccination became more cost-effective as the incidence rate and the vaccination rate increased.

Hagens et al. [31] estimated an ICER of vaccination over no vaccination at I$1249.9 from the Turkish health care system’s perspective for a I$23.9 vaccine with an effectiveness of 90% against disease and 45% against transmission, according to sensitivity analysis, the vaccine’s effectiveness and uptake have a profound impact on the cost-effectiveness of the vaccination campaign, while the price of each vaccine’s dose was responsible for only of 3.8% of the variability. 

According to the result of Padula et al. [29], the baseline strategy of not vaccinating would cost the U.S. health care system I$35 billion within a year, while the vaccination scenario would cost only I$13.5 billion and would prevent 3.4 million hospital days and 154,000 deaths. Thus, the immunization program was deemed as cost-saving when compared with no vaccination. According to the results of the probabilistic sensitivity analysis, which comprised 10,000 Monte Carlo simulations, vaccination has over a 60% probability of being sustainable at a cost-effectiveness threshold ranging from $50,000 to $500,000. Fernandes et al. [30] compared three different vaccines, namely, Oxford-AstraZeneca (Vaxzevria), Janssen, and CoronaVac to a baseline scenario without vaccination, the model simulated the effect of a vaccination campaign in a 100,000 individual cohort in Brazil. Vaxzevria and Janssen, which are both adenoviral vector vaccines, were cost-saving if compared to no vaccination, and the reported ICER were I$ −23,161.3 and I$ −1690.83, respectively. The ICER for the CoronaVac alternative was estimated at I$17,757.85.

## 4. Discussion

The present systematic review investigated the cost-effectiveness of COVID-19 vaccination campaigns over no vaccination. As shown by the DRM, all the selected studies demonstrated an unequivocal dominance of COVID-19 vaccination over no vaccination, the former being constantly cost-effective (i.e., below the WHO–CHOICE 3×GPD per capita threshold), if not cost-saving. The results of this study confirm that vaccination campaigns, as a preventive tool, are associated with cost-effective ICERs, as can be demonstrated by many studies published in the last 5 years and estimating the cost-effectiveness in different settings and scenarios than of the current pandemic. According to the findings of Pugh et al., the deployment of pneumococcal conjugate vaccines to an infant population would lead to an ICER ranging between I$308 per QALY and I$731 per QALY in Algeria; and between I$848 per QALY and I$1366 per QALY in Tunisia, depending on the type of vaccine employed. Based on the chosen cost-effectiveness threshold, which corresponds to the WHO–CHOICE 1×GPD per capita of each state, the vaccination campaigns were deemed highly cost-effective as the resulting ICERs fell below the thresholds [34]. These findings are also consistent with the ICER estimated by Rafferty et al. in Alberta Province, Canada, which fell between I$7704 per QALY and I$8137 per QALY, for chickenpox vaccination in children aged between 12 months and 6 years, on a health care system’s perspective, and was below the chosen cost-effectiveness threshold of $30,000 per QALY [35]. Routine childhood vaccination against seasonal influenza was cost-effective at I$3075.5 per QALY to EUR 1667.4 per QALY from a third-party payer’s perspective in Germany, as estimated by Scholz et al. [36]. A meta-analysis performed by Syeed et al. resulted in a pooled INB of I$53.49 (95% CI, 30.42–76.55) for low- and middle-income countries (LMIC) and of I$103.94 (95% CI, 75.28–132.60) for high-income countries for pneumococcal vaccination in children [37]. Quadrivalent influenza vaccination of the elderly population was cost-effective, according to the model of Jiang et al., if compared with no vaccination at a 29,580 threshold, as the ICER for the baseline scenario was I$6700 per QALY [36]. Yue et al. demonstrated that annual influenza vaccination of the elderly population was cost-effective in Singapore, although it could be cost-saving from a societal perspective if a proportion of the non-elderly is included in the vaccination program, for example, vaccinating all elderly and 20% of the rest of the population would lead to a negative ICER of −I$49,000 per QALY. The results of the above study suggest the impact of herd immunity on the sustainability of targeted vaccination campaigns [38]. 

Therefore, taking into consideration the supporting findings published in the scientific literature, this systematic review confirms that, also for the COVID-19 pandemic, mass vaccination campaigns can be considered not only as effective but also as sustainable and cost-effective tools to respond to a population-wide health threat. 

Three of the selected studies outlined the importance of an expeditious vaccine rollout, which is consistent with the findings of Bartsch et al., who estimated that achieving 50% coverage in 180 days in the U.S. with a 70% efficacious vaccine could prevent 20.9 million cases, 775,980 hospitalizations, and 91,660 deaths, and the loss of 977,730 QALYs with respect to achieving 40% coverage in 180 days [39].

Health systems are constantly searching for effective primary prevention strategies, and in recent years, several new vaccines have been produced such as those to counter the COVID-19 emergency in order to protect the population. However, to date, vaccine assessment includes only the basic information on efficacy, effectiveness, and safety needed for regulatory approval, rather than assessing the full public health value of vaccines [40]. Furthermore, vaccine efficacy and effectiveness usually focus on the direct protection of the vaccinated individual, but vaccination also prevents community outcomes through indirect protection, contributes to the sustainability of the health system through savings generated in terms of reductions in hospitalizations, direct medical costs, and any complications related to infectious diseases, and also supports health equity and national economies by reducing the productivity loss and safeguarding people’s health [40]. Therefore, economic evaluations of vaccines should also consider all these aspects and the full value of vaccinations. New data and evidence-based tools are also needed to support the decision-making process in the prevention field such as the value-based health care (VBHC) approach, in order to assess the full value of vaccinations [41]. The understanding of the whole value of vaccinations, and of prevention interventions in general, should be shared by all health actors and be oriented toward the goal of maximizing social well-being [42]. In fact, the concept of a value-based health system has recently been proposed as it is the whole health system that contributes to the well-being of society, thanks also to health promotion and prevention interventions [43]. Nonetheless, despite the effectiveness and cost-effectiveness of prevention interventions, investment in disease prevention remains low in many countries [42]. Among the barriers are the unwillingness to invest in actions that generally generate positive benefits in the long-term horizon and the difficulty of different actors to immediately enjoy the health benefits obtained from prevention [43]. Removing these barriers is necessary to improve the citizens’ health and the health system’s value, especially in priority areas for public health such as that of the control of infectious diseases. This vision is in line with the perspective of the Expert Panel on Effective Ways of Investing in Health (EXPH) of the European Commission (EC), which, in 2019, proposed a VBHC approach based on four value pillars: personal value, allocative value, technical value, and societal value [44]. The value concept proposed by the EXPH not only considers the costs and outcomes, but also the personal and social values associated with health care. According to this perspective, the guiding principles for a value-based health care system are access, equity, quality, performance, efficiency, and productivity (optimization and distribution of resources) [44]. These guiding principles should also be applied to the field of vaccine prevention. Even though vaccines contribute substantially to the reduction in the burden of infection diseases, estimating their value is extraordinarily complex. However, increasing the awareness of the full value of vaccinations is of great importance since hesitation and the underuse of vaccines may still lead to serious outbreaks [40]. In the prevention context, particular attention must be paid to COVID-19 vaccination, as COVID-19 represents a public health problem with a considerable impact from an epidemiological, clinical, economic, and societal point of view. Infectious diseases do not recognize geographical borders, but especially those preventable by vaccines such as COVID-19 require a global approach for their prevention and control. 

Moreover, policy makers must ensure their preparedness to the epidemic threats that the future may reserve by investing in vaccine research and development as well as by developing a sustainable infrastructure for the rapid production, distribution, and administration of the vaccines [45,46]. To secure a widespread adherence to the vaccination program, it is also important to tackle vaccine hesitancy through local community engagement [47]. From the perspective of global health, it is also paramount that policy makers facilitate the collaboration across countries by sharing scientific data and ensuring equitable access to vaccines from low-middle income countries, and as COVID-19 has demonstrated, viruses can easily spread along the routes of international trade [48]. Indeed, an equitable distribution of resources and universal access to vaccines through an international network of collaboration are also essential to help the recovery of the global economy from the unprecedented crisis that the current pandemic represents. The WHO is warning against the danger of vaccine hoarding, which could lead to the surge of novel SARS-CoV-2 strains capable of escaping immunization, and is urging policy makers to share vaccine doses with LMICs through the COVID-19 Vaccines Global Access (COVAX) network [49]. This initiative’s campaign involves a proportional framework to finance and distribute SARS-CoV-2 vaccines in LMICs, under the leadership of the Coalition for Epidemic Preparedness Innovations (CEPI), Gavi, and the WHO, with the participation of UNICEF as a key delivery partner. Furthermore, the protection against COVID-19 through vaccine or previous infection has been demonstrated to wane over time [50,51], thus COVID-19 vaccination campaigns might become ordinary practice in the years to come. 

The results of this systematic review should be assessed considering its main limitations and strengths. 

First, quite a small number of papers were retrieved and included for analysis. However, a robust evidence-based methodology was applied to conduct the present systematic review. 

Amongst the included studies, substantial heterogeneity could be noticed. Notwithstanding, the heterogeneity could be outlined by structural and methodological differences (i.e., modeling approach, time horizon, vaccine platform, vaccine efficacy, and the considered direct medical costs) across the analyzed studies. 

Moreover, another caveat is the cost variability among the analyzed papers. Nonetheless, this variance could be described by the scope of costs that each study adopted which, in turn, depends on the chosen perspective (i.e., patient, hospital, health care system, and societal). 

At the time of writing, it is still not clear how long natural immunity against reinfection can last, however, most authors have assumed that no reinfection occurred within the selected time frame, except for two studies [26,31]. Further research is thus required to investigate the cost-effectiveness of varying the value of the duration of natural immunity against reinfection in the economic model. Furthermore, there is also no scientific consensus about COVID-19 vaccine-induced protection against infection and transmission in a non-controlled setting and its duration over time [52], while its efficacy against severe disease is supported by solid evidence. Therefore, additional economic evaluations taking into account these two issues in the development of the economic model structure, the analysis of the data, and the reporting of the results are needed. Further research strictly assessing, from a health technology assessment perspective, the organizational impacts as well as the ethical, social, and legal aspects of COVID-19 vaccination campaigns is urgently required [53].

## 5. Conclusions

Although non-pharmaceutical interventions (e.g., lockdown) have demonstrated to be effective in curbing the spread of COVID-19 [54], vaccinations have been confirmed to be the most effective and sustainable public health measure, controlling the transmission of COVID-19 [55,56]. 

Based on what emerged from our review, there is a clear need to consider a value-based strategy of immunization against COVID-19. To do this, it is necessary to disseminate scientific evidence on the full value of COVID-19 vaccination, also in economic and cost-effective terms, in order to promote immunization strategies that consider the broader values of vaccination and to ensure access to high quality prevention for all citizens worldwide.

## Figures and Tables

**Table 2:** Quality assessment of the included economic evaluations.

Items	Wang et al. [23]	Debrabant et al. [24]	Jiang et al. [25]	Marco-Franco et al. [27]	Padula et al. [29]	Sandmann et al. [26]	Hagens et al. [31]	Kohli et al. [28]	Kirwin et al. [32]	Fernandes et al. [30]
1. Was a well-defined question posed in an answerable form?	Yes	Yes	Yes	Yes	Yes	Yes	Yes	Yes	Yes	Yes
2. Was a comprehensive description of the competing alternatives given (i.e., can you tell who did what to whom, where, and how often)?	No	No	No	No	No	Yes	No	No	Yes	No
3. Was the effectiveness of the program or services established?	Yes	Yes	Yes	Yes	No	No	Yes	No	No	Yes
4. Were all the important and relevant costs and consequences for each alternative identified?	No	No	No	No	Yes	Yes	Yes	No	Yes	Yes
5. Were costs and consequences measured accurately in appropriate physical units?	Yes	Yes	Yes	Yes	Yes	Yes	Yes	Yes	Yes	Yes
6. Were costs and consequences valued credibly?	Yes	Yes	Yes	Yes	Yes	Yes	Yes	Yes	Yes	Yes
7. Were costs and consequences adjusted for differential timing?	Unclear	Yes	Yes	Yes	No	Yes	Yes	Yes	Yes	Yes
8. Was an incremental analysis of costs and consequences of alternatives performed?	Yes	Yes	Yes	Yes	Yes	Yes	Yes	Yes	Yes	Yes
9. Was allowance made for uncertainty in the estimates of costs and consequences?	Yes	Yes	Yes	No	Yes	Yes	Yes	Yes	Yes	Yes
10. Did the presentation and discussion of study results include all issues of concern to users?	Yes	Unclear	Unclear	No	Yes	Yes	Unclear	No	No	Yes
Total score	7	7	7	6	7	9	8	6	8	9

**Table 3:** Three-by-three dominance ranking matrix.

First Author, Year, Country [Ref]	Costs *	Health Outcomes **	Judgement
Wang et al., 2021, Israel [23]	−	+	Favored
Debrabant et al., 2021, Denmark [24]	−	+	Favored
Jiang et al., 2022, Hong Kong, Indonesia, mainland China, Philippines, Singapore, and Thailand [25]	−	+	Favored
Sandmann et al., 2021, United Kingdom [26]	+	+	Unclear
Marco-Franco et al., 2021, Spain [27]	N/A	N/A	N/a
Hagens et al., 2021, Turkey [26]	+	+	Unclear
Kohli et al., 2021, United States [28]	+	+	Unclear
Kirwin et al., 2021, Canada [32]	N/A	N/A	N/a
Padula et al., 2021, United States [29]	−	+	Favored
Fernandes et al., 2022 † Brazil [30]	+	+	Unclear
Fernandes et al., 2022 ‡, Brazil [30]	−	+	Favored

* +: the vaccination is more costly than its alternative; −: the intervention is less costly that its alternative; ** +: the vaccination is more effective than its alternative; −: the vaccination is less effective than its alternative; † CoronaVac; ‡ Vaxzevria/Janssen.

## Data Availability

Data are available on reasonable request to the corresponding author.

## References

[1] WHO WHO Coronavirus (COVID-19) Dashboard. https://covid19.who.int/.

[2] World Bank (2020). Global Economic Prospects, June 2020.

[3] Coronavirus Disease (COVID-19): Vaccines. https://www.who.int/news-room/questions-and-answers/item/coronavirus-disease-(covid-19)-vaccines?gclid=CjwKCAiAz--OBhBIEiwAG1rIOgyiVjv0V25YyulXzGkzeHf0jwEHjPOvq5bBTiuv9M0g6o2GBLzxzxoCZB8QAvD_BwEtopicsurvey=v8kj13.

[4] The Different Types of COVID-19 Vaccines. https://www.who.int/news-room/feature-stories/detail/the-race-for-a-covid-19-vaccine-explained.

[5] Acosta-Coley I., Cervantes-Ceballos L., Tejeda-Benítez L., Sierra-Márquez L., Cabarcas-Montalvo M., García-Espiñeira M., Coronell-Rodríguez W., Arroyo-Salgado B. (2022). Vaccines Platforms and COVID-19: What You Need to Know. Trop. Dis. Travel Med. Vaccines.

[6] Sridhar S., Brokstad K., Cox R. (2015). Influenza Vaccination Strategies: Comparing Inactivated and Live Attenuated Influenza Vaccines. Vaccines.

[7] Wang N., Shang J., Jiang S., Du L. (2020). Subunit Vaccines Against Emerging Pathogenic Human Coronaviruses. Front. Microbiol..

[8] van Riel D., de Wit E. (2020). Next-Generation Vaccine Platforms for COVID-19. Nat. Mater..

[9] University of Oxford Coronavirus (COVID-19) Vaccinations. Our World in Data..

[10] Africa Clocks Fastest Surge in COVID-19 Cases This Year, but Deaths Remain Low. WHO Africa. https://www.afro.who.int/news/africa-clocks-fastest-surge-covid-19-cases-year-deaths-remain-low.

[11] Drummond M. (2015). Methods for the Economic Evaluation of Health Care Programmes.

[12] World Health Organization (2019). WHO Guide for Standardization of Economic Evaluations of Immunization Programmes.

[13] Hoch J.S., Dewa C.S. (2008). A Clinician’s Guide to Correct Cost-Effectiveness Analysis: Think Incremental Not Average. Can. J. Psychiatry.

[14] Ramsey S., Willke R., Briggs A., Brown R., Buxton M., Chawla A., Cook J., Glick H., Liljas B., Petitti D. (2005). Good Research Practices for Cost-Effectiveness Analysis Alongside Clinical Trials: The ISPOR RCT-CEA Task Force Report. Value Health.

[15] Elliott R., Payne K. (2005). Essentials of Economic Evaluation in Healthcare.

[16] Willan A.R. (2004). Incremental Net Benefit in the Analysis of Economic Data from Clinical Trials, with Application to the CADET-Hp Trial. Eur. J. Gastroenterol. Hepatol..

[17] Stinnett A.A., Mullahy J. (1998). Net Health Benefits: A New Framework for the Analysis of Uncertainty in Cost-Effectiveness Analysis. Med. Decis. Making.

[18] Doran C.M. (2008). Economic Evaluation of Interventions to Treat Opiate Dependence: A Review of the Evidence. PharmacoEconomics.

[19] International Monetary Fund Consumer Price Index (CPI). https://data.imf.org/?sk=4FFB52B2-3653-409A-B471-D47B46D904B5&sId=1485878855236.

[20] OECD (2022). Purchasing Power Parities (PPP). https://data.oecd.org/conversion/purchasing-power-parities-ppp.htm.

[21] The Joanna Briggs Institute The Joanna Briggs Institute Joanna Briggs Institute Reviewers’ Manual. https://nursing.lsuhsc.edu/JBI/docs/ReviewersManuals/Economic.pdf.

[22] Iino H., Hashiguchi M., Hori S. (2022). Estimating the Range of Incremental Cost-Effectiveness Thresholds for Healthcare Based on Willingness to Pay and GDP per Capita: A Systematic Review. PLoS ONE.

[23] Wang W.-C., Fann J.C.-Y., Chang R.-E., Jeng Y.-C., Hsu C.-Y., Chen H.-H., Liu J.-T., Yen A.M.-F. (2021). Economic Evaluation for Mass Vaccination against COVID-19. J. Formos. Med. Assoc..

[24] Debrabant K., Grønbæk L., Kronborg C. (2021). The Cost-Effectiveness of a COVID-19 Vaccine in a Danish Context. Clin. Drug Investig..

[25] Jiang Y., Cai D., Shi S. (2022). Economic Evaluations of Inactivated COVID-19 Vaccines in Six Western Pacific and South East Asian Countries and Regions: A Modeling Study. Infect. Dis. Model..

[26] Sandmann F.G., Davies N.G., Vassall A., Edmunds W.J., Jit M., Sun F.Y., Villabona-Arenas C.J., Nightingale E.S., Showering A., Knight G.M. (2021). The Potential Health and Economic Value of SARS-CoV-2 Vaccination alongside Physical Distancing in the UK: A Transmission Model-Based Future Scenario Analysis and Economic Evaluation. Lancet Infect. Dis..

[27] Marco-Franco J.E., Pita-Barros P., González-de-Julián S., Sabat I., Vivas-Consuelo D. (2021). Simplified Mathematical Modelling of Uncertainty: Cost-Effectiveness of COVID-19 Vaccines in Spain. Mathematics.

[28] Kohli M., Maschio M., Becker D., Weinstein M.C. (2021). The Potential Public Health and Economic Value of a Hypothetical COVID-19 Vaccine in the United States: Use of Cost-Effectiveness Modeling to Inform Vaccination Prioritization. Vaccine.

[29] Padula W.V., Malaviya S., Reid N.M., Cohen B.G., Chingcuanco F., Ballreich J., Tierce J., Alexander G.C. (2021). Economic Value of Vaccines to Address the COVID-19 Pandemic: A U.S. Cost-Effectiveness and Budget Impact Analysis. J. Med. Econ..

[30] Fernandes R.R.A., da Silva Santos M., da Silva Magliano C.A., Tura B.R., Macedo L.S.D.N., Padila M.P., França A.C.W., Braga A.A. (2022). Cost Utility of Vaccination Against COVID-19 in Brazil. Value Health Reg. Issues.

[31] Hagens A., İnkaya A.Ç., Yildirak K., Sancar M., van der Schans J., Acar Sancar A., Ünal S., Postma M., Yeğenoğlu S. (2021). COVID-19 Vaccination Scenarios: A Cost-Effectiveness Analysis for Turkey. Vaccines.

[32] Kirwin E., Rafferty E., Harback K., Round J., McCabe C. (2021). A Net Benefit Approach for the Optimal Allocation of a COVID-19 Vaccine. PharmacoEconomics.

[33] WHO Regional Offices World Health Organizaton. https://www.who.int/about/who-we-are/regional-offices.

[34] Pugh S.J., Fletcher M.A., Charos A., Imekraz L., Wasserman M., Farkouh R. (2019). Cost-Effectiveness of the Pneumococcal Conjugate Vaccine (10- or 13-Valent) Versus No Vaccination for a National Immunization Program in Tunisia or Algeria. Infect. Dis. Ther..

[35] Rafferty E.R.S., McDonald W., Osgood N.D., Doroshenko A., Farag M. (2021). What We Know Now: An Economic Evaluation of Chickenpox Vaccination and Dose Timing Using an Agent-Based Model. Value Health.

[36] Scholz S.M., Weidemann F., Damm O., Ultsch B., Greiner W., Wichmann O. (2021). Cost-Effectiveness of Routine Childhood Vaccination Against Seasonal Influenza in Germany. Value Health.

[37] Syeed M.S., Ghule P., Le L.M., Veettil S.K., Horn E.K., Perdrizet J., Wasserman M., Thakkinstian A., Chaiyakunapruk N. (2022). Pneumococcal Vaccination in Children: A Systematic Review and Meta-Analysis of Cost-Effectiveness Studies. Value Health.

[38] Yue M., Dickens B.L., Yoong J.S., I-Cheng Chen M., Teerawattananon Y., Cook A.R. (2019). Cost-Effectiveness Analysis for Influenza Vaccination Coverage and Timing in Tropical and Subtropical Climate Settings: A Modeling Study. Value Health.

[39] Bartsch S.M., Wedlock P.T., O’Shea K.J., Cox S.N., Strych U., Nuzzo J.B., Ferguson M.C., Bottazzi M.E., Siegmund S.S., Hotez P.J. (2021). Lives and Costs Saved by Expanding and Expediting Coronavirus Disease 2019 Vaccination. J. Infect. Dis..

[40] Calabro’ G.E., Carini E., Tognetto A., Giacchetta I., Bonanno E., Mariani M., Ricciardi W., de Waure C. (2022). The Value(s) of Vaccination: Building the Scientific Evidence According to a Value-Based Healthcare Approach. Front. Public Health.

[41] de Waure C., Calabrò G.E., Ricciardi W. (2022). Recommendations to Drive a Value-Based Decision-Making on Vaccination. Expert Rev. Vaccines.

[42] World Health Organization Regional Office for Europe, European Observatory on Health Systems and Policies & McDaid, D. Using Economic Evidence to Help Make the Case for Investing in Health Promotion and Disease Prevention.

[43] Smith P.C., Sagan A., Siciliani L., Panteli D., McKee M., Soucat A., Figueras J. (2020). Building on Value-Based Health Care: Towards a Health System Perspective.

[44] European Commission (2019). Directorate General for Health and Food Safety. Defining Value in ‘Value-Based Healthcare’: Report of the Expert Panel on Effective Ways of Investing in Health (EXPH).

[45] Gross D.P., Sampat B.N. (2021). The Economics of Crisis Innovation Policy: A Historical Perspective. AEA Pap. Proc..

[46] Hodes S., Majeed A. (2021). Building a Sustainable Infrastructure for Covid-19 Vaccinations Long Term. BMJ.

[47] Majeed A., Pollock K., Hodes S., Papaluca M. (2022). Implementation of Covid-19 Vaccination in the United Kingdom. BMJ.

[48] Deb P., Furceri D., Jimenez D., Kothari S., Ostry J.D., Tawk N. (2022). The Effects of COVID-19 Vaccines on Economic Activity. Swiss J. Econ. Stat..

[49] Working for Global Equitable Access to COVID-19 Vaccines. COVAX. https://www.who.int/initiatives/act-accelerator/covax.

[50] Goldberg Y., Mandel M., Bar-On Y.M., Bodenheimer O., Freedman L.S., Ash N., Alroy-Preis S., Huppert A., Milo R. (2022). Protection and Waning of Natural and Hybrid Immunity to SARS-CoV-2. N. Engl. J. Med..

[51] Menni C., May A., Polidori L., Louca P., Wolf J., Capdevila J., Hu C., Ourselin S., Steves C.J., Valdes A.M. (2022). COVID-19 Vaccine Waning and Effectiveness and Side-Effects of Boosters: A Prospective Community Study from the ZOE COVID Study. Lancet Infect. Dis..

[52] Franco-Paredes C. (2022). Transmissibility of SARS-CoV-2 among Fully Vaccinated Individuals. Lancet Infect. Dis..

[53] Revealing the Hidden Value of Vaccines. https://www.nature.com/articles/d42473-021-00520-w.

[54] Gianino M.M., Nurchis M.C., Politano G., Rousset S., Damiani G. (2021). Evaluation of the Strategies to Control COVID-19 Pandemic in Four European Countries. Front. Public Health.

[55] Mohammed I., Nauman A., Paul P., Ganesan S., Chen K.-H., Jalil S.M.S., Jaouni S.H., Kawas H., Khan W.A., Vattoth A.L. (2022). The Efficacy and Effectiveness of the COVID-19 Vaccines in Reducing Infection, Severity, Hospitalization, and Mortality: A Systematic Review. Hum. Vaccines Immunother..

[56] Thompson J., Wattam S. (2021). Estimating the Impact of Interventions against COVID-19: From Lockdown to Vaccination. PLoS ONE.

